# Enhanced Pseudo-Capacitive Contributions to High-Performance Sodium Storage in TiO_2_/C Nanofibers via Double Effects of Sulfur Modification

**DOI:** 10.1007/s40820-020-00506-1

**Published:** 2020-08-14

**Authors:** Yan Zhang, Yuanye Huang, Vesna Srot, Peter A. van Aken, Joachim Maier, Yan Yu

**Affiliations:** 1 Max Planck Institute for Solid State Research, Stuttgart, 70569 Germany; 2 Hefei National Laboratory for Physical Sciences at the Microscale, Department of Materials Science and Engineering, Key Laboratory of Materials for Energy Conversion, Chinese Academy of Sciences (CAS), University of Science and Technology of China, Hefei, 230026 Anhui People’s Republic of China; 3 Dalian National Laboratory for Clean Energy (DNL), Chinese Academy of Sciences (CAS), Dalian, 116023 Liaoning People’s Republic of China

**Keywords:** Sodium-ion battery, Pseudo-capacitive, Anodes, TiO_2_/C nanofibers, Sulfur doped

## Abstract

Pseudo-capacitive mechanisms can provide higher energy densities than electrical double-layer capacitors while being faster than bulk storage mechanisms. Usually, they suffer from low intrinsic electronic and ion conductivities of the active materials. Here, taking advantage of the combination of TiS_2_ decoration, sulfur doping, and a nanometer-sized structure, as-spun TiO_2_/C nanofiber composites are developed that enable rapid transport of sodium ions and electrons, and exhibit enhanced pseudo-capacitively dominated capacities. At a scan rate of 0.5 mV s^−1^, a high pseudo-capacitive contribution (76% of the total storage) is obtained for the S-doped TiS_2_/TiO_2_/C electrode (termed as TiS_2_/S-TiO_2_/C). Such enhanced pseudo-capacitive activity allows rapid chemical kinetics and significantly improves the high-rate sodium storage performance of TiO_2_. The TiS_2_/S-TiO_2_/C composite electrode delivers a high capacity of 114 mAh g^−1^ at a current density of 5000 mA g^−1^. The capacity maintains at high level (161 mAh g^−1^) even after 1500 cycles and is still characterized by 58 mAh g^−1^ at the extreme condition of 10,000 mA g^−1^ after 10,000 cycles.

**Electronic supplementary material:**

The online version of this article (10.1007/s40820-020-00506-1) contains supplementary material, which is available to authorized users.

## Highlights

- One-dimensional elongated TiS_2_-modified and S-doped TiO_2_/C nanofibers electrode was synthesized through electrospinning, which exhibited a high specific capacity, excellent cyclic stability, and rate capability in sodium-ion battery.
- An enhanced pseudo-capacitive capacity because of S doping and TiS_2_ decoration contributes to noticeable sodium storage performance. High capacity of 161 mAh g^−1^ (at 3000 mA g^−1^) after 1500 cycles and 58 mAh g^−1^ (at 10,000 mA g^−1^) after 10,000 cycles is delivered outstandingly.

**Electronic supplementary material:**

The online version of this article (10.1007/s40820-020-00506-1) contains supplementary material, which is available to authorized users.

## Graphical abstract

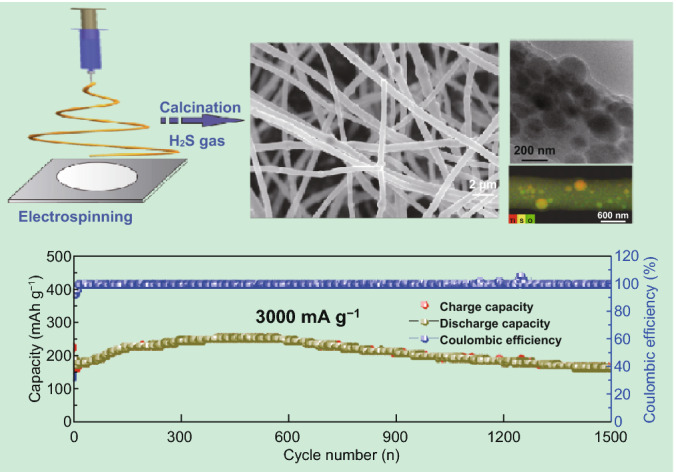

## Introduction

Sodium-ion batteries (SIBs) are currently considered as large-scale energy storage systems potentially replacing lithium-ion batteries (LIBs) because of greater abundance and of lower cost [1, 2]. Even though the larger ion radius and higher molar mass appear to be kinetically and thermodynamically disadvantageous, the lower polarization exerted by Na^+^ possesses advantages in terms of ion mobility and charge-transfer kinetics. In spite of the homologous role in the periodic system, the working chemistry of SIBs is not directly adaptable from LIBs [3, 4]. Graphitic carbon, as anode in the commercial LIBs (a capacity of 372 mAh g^−1^), stores sodium only with a specific capacity of 31 mAh g^−1^ [5, 6]. As reported, doped graphite with enlarged layer distances (more than 0.37 nm) has been proposed as a rational solution, to more easily accommodate sodium ions between the parallel graphene sheets [7–12]. Introducing heteroatoms into the active material has also been suggested to be an effective way to promote the sodium storage performance of titanium-based materials [13–15]. For example, C-, N-doped titanium dioxide (TiO_2_) can exhibit a remarkable sodium storage capacity [16]. The introduction of heteroatoms influences the thermodynamic and kinetic features by their sheer presence as well as their doping effect.

Recently, for titanium-based materials, pseudo-capacitive charge storage mechanisms have been shown to increase the overall energy storage behavior. For instance, previous work by Lukatskaya et al. showed ultrahigh-rate pseudo-capacitive energy storage in 2D Ti_3_C_2_T_x_ materials (T denotes surface terminations) [17]. The TiO_2_-based insertion materials are considered as the most promising SIBs anodes [18]. Pseudo-capacitive sodium storage in a composite of nanostructured doped TiO_2_ with graphene has been reported to be characterized by high-rate performance [19–21], which is enabled by improved charge transfer and electron/ion conductivities [22].

Graphene-analogous transition metal dichalcogenides (TMDC), such as MoS_2_, VS_2_, MoSe_2_, TiS_2_, NiS_2_, and CoS_2_, have been also regarded as relevant pseudo-capacitive materials with potentially improved energy storage [23]. Among these, TiS_2_ is the lightest and cheapest, and owing to the lower electronegativity of sulfur, it is less ionic than TiO_2_. It has been proposed as sodium storage energy system by Winn et al. [24] and by Newman et al. [25]. It exhibits exceedingly high electronic conductivities (10^3^ ohm^−1^ cm^−1^) [26], substantial ion diffusion rates [27], low volume expansion as well as the absence of phase change during cycling.

These assets let us develop TiS_2_-modified S-doped TiO_2_/C nanofibers composites (referred as TiS_2_/S-TiO_2_/C) via electrospinning, where the carbon nanofiber acts as framework, and TiS_2_ as pseudo-capacitive decoration of the TiO_2_ matrix. Owing to the combination of helpful morphological and compositional features, the TiS_2_/S-TiO_2_/C nanofibers exhibit a superb high-rate performance and long-term cycling life in SIBs.

## Experimental Section

### Materials Preparation

For electrospinning, 1.0 g polyacrylonitrile (PAN, Mw = 150,000, Sigma-Aldrich) was firstly dissolved in 9 mL N,N-dimethylformamide (DMF) at room temperature through vigorous stirring. Then, 1.5 mL titanium (IV) butoxide (TBOT, liquid, Sigma-Aldrich) was added to the above solution. A homogeneous precursor solution for electrospinning was prepared under strong stirring for 12 h. The resultant precursor solution was poured into a 5 mL plastic syringe connected to an 18-gauge blunt tip needle. The solution flow rate of 1 mL h^−1^ is adjusted by a syringe pump (New Era, Era-1000, USA). A voltage of 19 kV was provided by a high voltage–power supply (Model HCE35-35000, FUG DC power source, Germany). An aluminum foil was placed 15 cm below the needle as collector of the products. The as-obtained PAN-TBOT membrane was directly carbonized at 700 °C for 3 h with a heating rate of 5 °C min^−1^ in different gas atmospheres. The samples obtained in H_2_S or argon gas were termed as TiS_2_/S-TiO_2_/C or TiO_2_/C.

### Materials Characterization

The crystal phase of composition was measured by XRD on a Philips PW 3020 machine with Cu Kα radiation. The structures and morphology were observed on JEOL 6300F field-emission scanning electron microscope (FESEM, Tokyo, Japan) at 15 kV. HRTEM and HAADF-STEM imaging combined with analytical EDX measurements were carried out at 200 kV with an advanced TEM (JEOL ARM200F, JEOL Co. Ltd.), equipped with a cold field-emission gun and a CETCOR image corrector (CEOS Co. Ltd.). XPS measurements were performed with a Kratos Axis Ultra instrument using monochromatized Al kα X-rays. (Kratos Analytical Ltd, UK). Thermogravimetric analysis was undertaken on a thermal analysis instrument (NETZSCH STA449F3, Germany). The Brunauer–Emmett–Teller (BET, BELSORP-MINI II) specific surface area was calculated according to the nitrogen adsorption–desorption curves at 77 K. The pore size distribution was determined on the basis of the Barrett–Joyner–Halenda (BJH) method.

### Electrochemical Measurements

The homogenous mix slurry was made of active materials (TiS_2_/S-TiO_2_/C or TiO_2_/C), Super-P (carbon black, Timcal), and carboxymethyl cellulose (CMC, Sigma-Aldrich) binder with a weight ratio of 70:15:15. The as-prepared slurry was pasted on a copper current collector and dried in a vacuum oven for 12 h at 60 °C. In CR2032-type coin cell, the coated copper disks were used as working electrode, sodium metal foil was used as counter/reference electrode, 1 M solution of NaClO_4_ (Sigma-Aldrich, 99%) in a 95:5 vol/vol mixture of propylene carbonate (PC, Sigma-Aldrich, anhydrous 99.7%) and fluoroethylene carbonate (FEC, Sigma-Aldrich, 99%) was used as electrolyte, and a glass fiber (GF/D) was used as separator. The assembling of sodium-ion battery was finished in a MBraun glove box filled with highly pure argon gas (O_2_ and H_2_O levels < 0.5 ppm). The half-cell was galvanostatically discharged and charged in BTS battery test instrument (Neware BTS 7.0, Shenzhen). Cyclic voltammetry (CV) measurements were performed on Autolab instruments at various scan rates. These measurements were performed in the fixed voltage window between 3.0 and 0.01 V.

## Results and Discussion

### Crystallographic and Morphological Structure

**Scheme 1:**
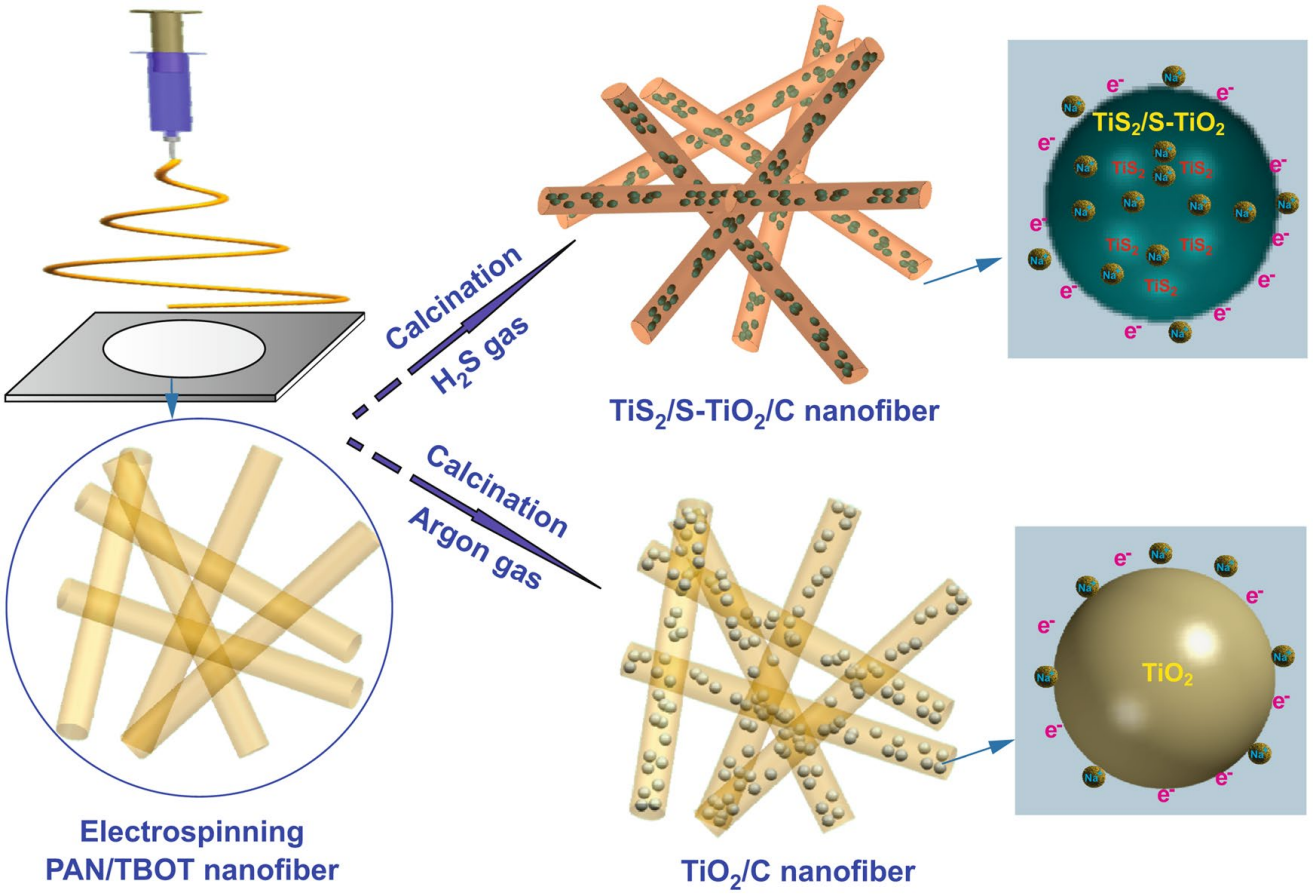
Schematic illustrations of the synthesis process for TiS_2_/S-TiO_2_/C nanofiber and TiO_2_/C nanofibers

**Fig. 1:**
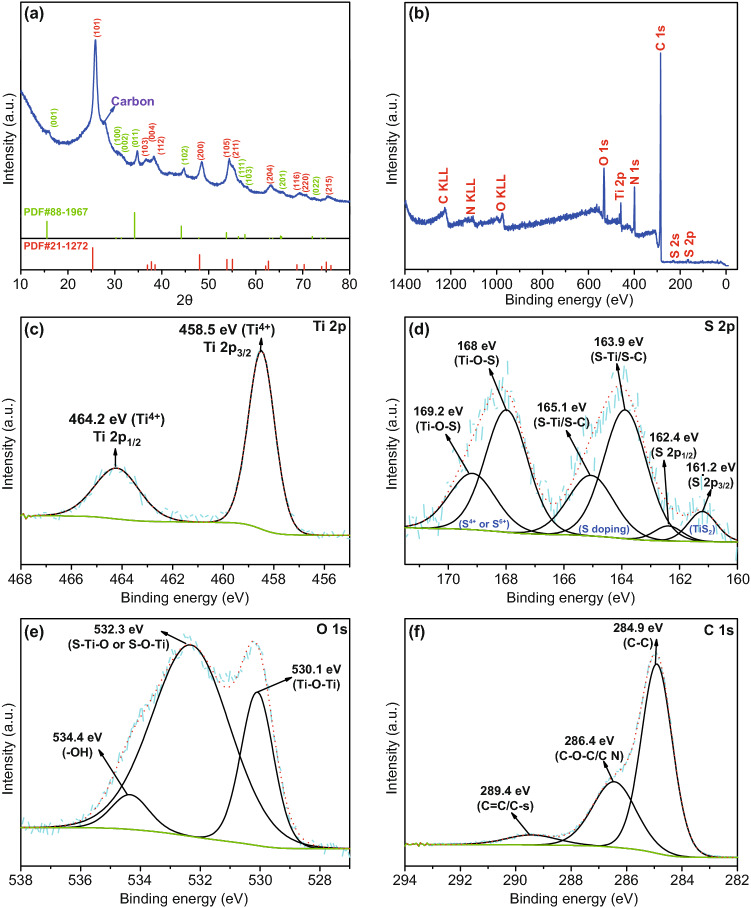
**a** XRD patterns. **b** Integrated XPS spectrum. **c**–**f** High-resolution XPS spectra of Ti 2p, S 2p, O 1 s, and C 1 s of the as spun of TiS_2_/S-TiO_2_/C nanofibers composites

As shown in Scheme 1, TiS_2_/S-TiO_2_/C nanofibers were prepared by electrospinning followed by annealing of PAN-tetrabutyl orthotitanate nanofibers (termed as PAN-TBOT) precursors in an H_2_S atmosphere. TiO_2_/C nanofibers without sulfur components were obtained through sintering of same precursors fibers under argon gas. For TiS_2_/S-TiO_2_/C nanofibers, 1D nanofiber structure can provide a fast electron pathway and alleviate the volume stress; the synergistic effects of the sulfur doping and TiS_2_ decoration enable fast ion/electron transfer in the TiO_2_ phase and improve the interfacial storage. The XRD patterns (Figs. 1a and S1) show two samples with a typical crystalline structure of the anatase phase (PDF#21-1272); three obvious peaks at 16.0°, 34.8°, and 44.6° are only observed in the TiS_2_/S-TiO_2_/C sample, corresponding to the (001), (011), and (102) crystal planes of TiS_2_, respectively (PDF#88-1967) [28, 29]. In Figs. 1 and S1, the broad and small peaks at about 26° correspond to the carbon content (marked by arrows). X-ray photoelectron spectroscopy (XPS) was performed to verify the chemical state of TiS_2_/S-TiO_2_/C nanofibers (Fig. 1b–f). The integral survey spectrum of TiS_2_/S-TiO_2_/C nanofibers shows O, Ti, C, N, and S components. Regarding the Ti 2p peaks, the two peaks at 458.5 and 464.2 eV are assigned to Ti 2p_3/2_ and Ti 2p_1/2_ of the tetravalent Ti ion (Fig. 1c). In the high-resolution spectrum of S 2p (Fig. 1d), the chemical state of sulfur was characterized by several peaks. The low-energy ones at 161.2 and 162.4 eV can be attributed to the TiS_2_ phase [30, 31]. The high-energy doublet centered at 163.9 and 165.1 eV can be correlated with S-Ti and S-C, which confirm S doping of TiO_2_ bulk and of carbon fibers [32]. It is possible that the calcination in H_2_S flow results in partial substitution of O by S atoms. The sulfur peaks of strong intensity at 167–170 eV suggest that sulfur replaces Ti^4+^ in the form of S^4+^ or S^6+^ [32, 33]. In line with a previous report by Devi et al., the replacement of Ti by sulfur leads to the formation of Ti–O–S bonds and is more favorable than the substitution of O^2−^ by S^2−^ [34]. The O 1 s signal at 534.4 eV also confirms the presence of Ti–O–S bond (Fig. 1e). The C 1 s features are assignable to C–C (284.9 eV), C=O or C=N (286.4 eV), and C–O–C or C–S bonds (289.4 eV) (Fig. 1f), which stem from the polyacrylonitrile (PAN) polymer precursor. These XPS results demonstrate the existence of TiS_2_ and S defects within the TiO_2_ lattice, which is expected to improve the sodium storage performance of TiS_2_/S-TiO_2_/C composites.

**Fig. 2:**
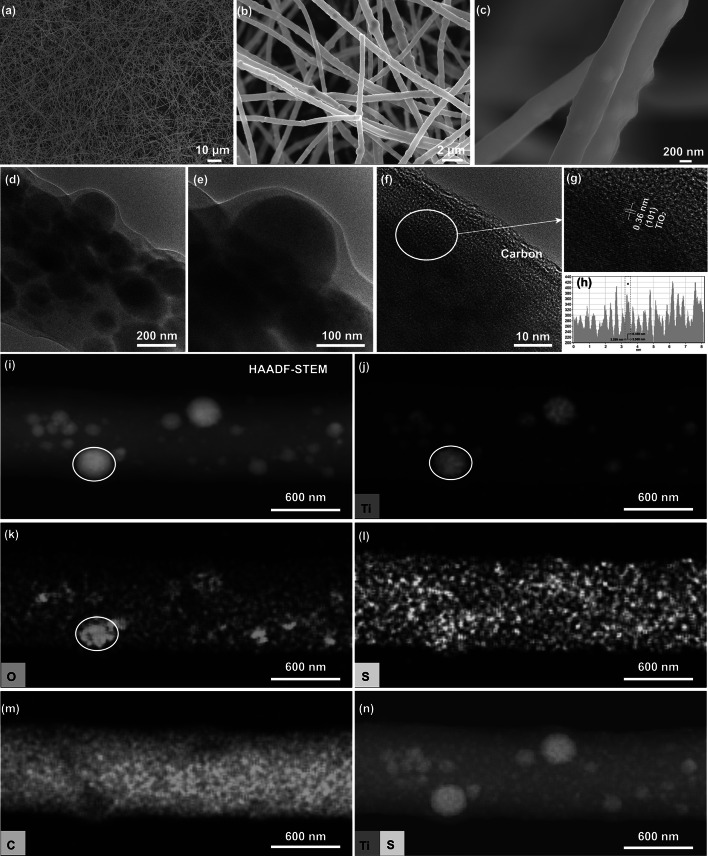
**a**–**c** SEM images. **d**–**e** BF-TEM images. **f**–**h** Representative HRTEM image with indicated measured distances between the lattice planes in crystalline TiO_2_ core. **i** HAADF-STEM image with of as-spun TiS_2_/S-TiO_2_/C single nanofiber with the corresponding **j** Ti, **k** O, **l** S, **m** C, and **n** overlay of Ti and S EDX maps

The morphology and structure of as-obtained TiS_2_/S-TiO_2_/C nanofibers are shown in Fig. 2a, b, displaying a well-dispersed and interwoven fibrous network, which is similar with the structure of PAN-TBOT precursors (Fig. S2a, b) and TiO_2_/C nanofibers (Fig. S2c, d). At a larger magnification, 1D nanofibers display a uniform diameter distribution of about 600 nm, whereby some nanoparticles are aggregated inside the fibers (Fig. 2c). The bright-field (BF) transmission electron microscope (TEM) images (Fig. 2d, e) visualize these particles with sizes of several ten nanometers. In the high-resolution TEM (HRTEM) (Fig. 2f, g), the measured lattice fringe spacing of 0.36 nm corresponds to the (101) lattice plane spacing of anatase TiO_2_. The amorphous regions around the lattice fringes are ascribed to the carbon phase. The carbon nanofiber may help to improve the mechanical stability for long-term cycle operation and can serve as lead for electron transfer throughout the overall electrode. High-angle annular dark-field (HAADF) scanning TEM (STEM) image of a representative TiS_2_/S-TiO_2_/C single nanofiber (Fig. 2i) and corresponding Ti, O, S, C (Fig. 2j–m) and overlapped Ti/S EDX elemental maps (Fig. 2n) are presented. The Ti and O EDX maps clearly exhibit enriched regions at the position of particles inside the fibers. Although sulfur is quite homogenously distributed within the nanofibers, S mapping exhibits a slightly enhanced concentration in the regions around the particles. Additionally, the higher signals of S, Ti, and O in the particles from the elemental maps (Fig. 2j–l) together with the XRD and XPS results confirm the presence of TiS_2_ and sulfur-doped TiO_2_. The nitrogen adsorption–desorption isotherm was measured to determine the Brunauer–Emmett–Teller (BET) surface area and pore size distribution, as shown in Fig. S3a, b. It showed that the specific surface area and the average pore size of TiS_2_/S-TiO_2_/C nanofibers were 146.1 m^2^ g^−1^ and 12–17 nm, respectively, which is ascribed to the interwoven fibrous network affording a large surface area and porous structure.

### Electrochemical Properties

**Fig. 3:**
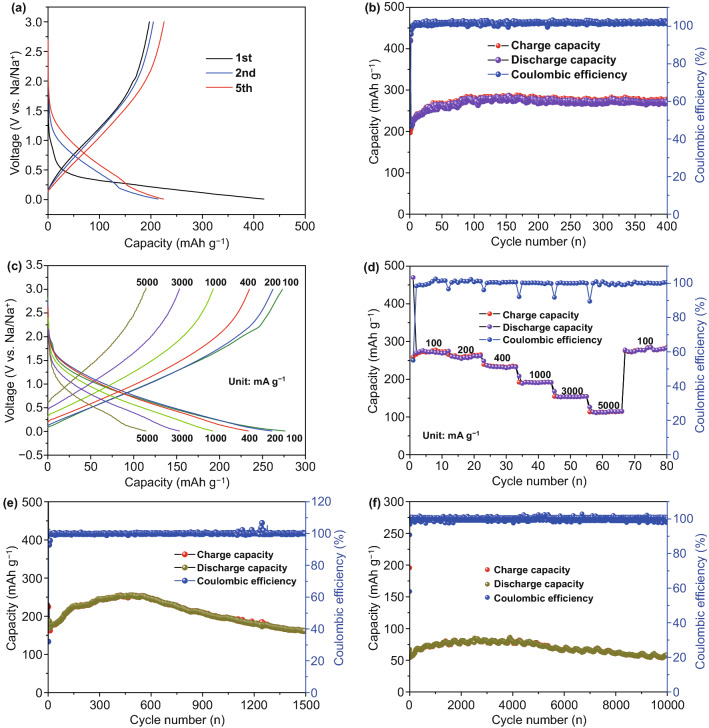
**a** Charge–discharge profiles of the first, second, and fifth cycle. **b** Cycling performances at a current density of 100 mA g^−1^. **c** Charge–discharge profiles at different rates. **d** Rate performances. **e** Cycling performance at a rate of 100 mA g^−1^ for four cycles and a rate of 3000 mA g^−1^ for 1500 times. **f** Long-term cycling performance at a rate of 100 mA g^−1^ for four cycles and a rate of 10,000 mA g^−1^ for 10,000 times of TiS_2_/S-TiO_2_/C nanofibers electrode in SIBs

To explore the influence of sulfur doping and TiS_2_ modification on the sodium storage performance of TiO_2_/C electrode, the electrochemical performance of as-spun TiS_2_/S-TiO_2_/C nanofibers composites, TiO_2_/C nanofibers composites, and pure C nanofibers as anodes in SIBs was investigated. All of the electrochemical measurements were performed at voltages of 0.01–3.0 V. Figure 3a displays the discharge–charge profile in the first five cycles for the TiS_2_/S-TiO_2_/C electrode at a current density of 100 mA g^−1^. In the first discharge–charge process, the TiS_2_/S-TiO_2_/C electrode delivers an initial discharge-specific capacity of 419.6 mAh g^−1^ and charge-specific capacity of 197.2 mAh g^−1^, which are higher than those of the TiO_2_/C electrode (Fig. S4a, discharge-specific capacity: 330.7 mAh g^−1^, charge-specific capacity: 159.8 mAh g^−1^). Both electrodes show irreversible capacities in the first cycle, which is ascribed to the formation of a solid electrolyte interface (SEI) layer on the electrode surface and the decomposition of the electrolyte [35]. When the voltage is below 1.5 V, the two electrodes show similar voltage slopes and working voltages profile, strongly suggesting that the sodium storage contribution mainly stemmed from the TiO_2_ component. Notably, a characteristic charge plateau located at higher working voltages in the range of 1.5–2.5 V is attributed to sodium extraction out of TiS_2_ materials [36]. After 400 cycles, the reversible capacity of TiS_2_/S-TiO_2_/C composites electrode maintained at 274 mAh g^−1^ (Fig. 3b), which is markedly superior to 199 mAh g^−1^ of TiO_2_/C composites electrode (Fig. S4b, 400th cycle) and about 162 mAh g^−1^ of pure C nanofiber electrode (Fig. S5, 50th cycle). After three cycles, the high Coulombic efficiency is close to 100%, demonstrating the cycling reversibility of TiS_2_/S-TiO_2_/C electrode. Notably, good cyclability for TiS_2_/S-TiO_2_/C composites electrode and TiO_2_/C composites electrode is closely connected with the unique structure of interwoven fibrous network with assembling nanoparticles in a nanofiber, which can be proved the morphologies after discharged and charged cycling (Fig. S8).

As plotted in Fig. 3c, d, the rate performances of the electrodes are further evaluated at different current densities from 100 to 5000 mA g^−1^. When increasing the current density, the TiS_2_/S-TiO_2_/C electrode exhibits 271.4 mAh g^−1^ (100 mA g^−1^), 258.7 mAh g^−1^ (200 mA g^−1^), 235.5 mAh g^−1^ (400 mA g^−1^), 191.6 mAh g^−1^ (1000 mA g^−1^), 153.8 mAh g^−1^ (3000 mA g^−1^), and 114.2 mAh g^−1^ (5000 mA g^−1^), respectively, whereas the TiO_2_/C electrode shows only 210.1, 175.4, 145.3, 111.9, 70.16, and 40.7 mAh g^−1^ at the corresponding rates in Fig. S4c. When the current density is set back to 100 mA g^−1^, the specific capacity of the TiS_2_/S-TiO_2_/C electrode can recover to 284.9 mAh g^−1^, indicating an outstanding rate capability. The rate performance of the TiS_2_/S-TiO_2_/C electrode is superior to that of the TiO_2_/C electrode in the entire rate range, showing the improvement of the electrochemical activity of TiO_2_ with the help of the introduction of TiS_2_ and S doping. When the TiS_2_/S-TiO_2_/C electrode is cycled in 3000 mA g^−1^ for 1500 cycles, it still exhibits a high capacity of 161 mAh g^−1^ (Fig. 3e). Most impressively, a stable capacity of 58 mAh g^−1^ at ultrahigh current density of 10,000 mA g^−1^ (Fig. 3f) is maintained after 10,000 cycles, indicating excellent ultralong cycling stability. To the best of our knowledge, the TiS_2_/S-TiO_2_/C nanofiber electrode is comparable to the best performances reported as-spun TiO_2_ nanofiber anode in the literature in terms of both cycling stability and rate capability (Table 1). The improvements of the electrochemistry performance may be attributed to promoted interfacial charge-transfer kinetics of high conductivity TiS_2_ and increased sodium storage sites at the TiS_2_/TiO_2_ grain boundaries. Moreover, the carbon nanofibers may help the mechanical stability and the electron/ion diffusivity, which synergistically improve electrochemical activity of TiO_2_ at high rate.

**Table 1:** Comparison of the sodium storage performances of as-spun TiS_2_/S-TiO_2_/C nanofibers with previously reported electrospun TiO_2_ nanofiber anode in SIBs

Materials	High-rate capacity (mAh g^−1^)	Cycle performances (mAh g^−1^)	Initial Coulombic efficiency	Publication years
TiO_2_/C nanofibers [46]	164.9 at 2000 mA g^−1^	237.1 at 200 mA g^−1^ over 1000 cycles;	58%	2016
N-doped anatase TiO_2_ nanofibers [47]	110 at 3350 mA g^−1^	110 at 3350 mA g^−1^ over 500 cycles;	/	2016
TiO_2−x_ nanocages anchored carbon fiber [48]	120 at 1000 mA g^−1^	150 at 1000 mA g^−1^ over 1000 cycles;	38.3%	2018
Amorphous black TiO_2-x_/C nanofiber [49]	61 at 415 mA g^−1^	90 at 16.6 mA g^−1^ over 100 cycles;	/	2018
TiS_2_/S-TiO_2_/C nanofibers (this work)	114.2 at 5000 mA g^−1^	161 at 3000 mA g^−1^ over 1500 cycles; 58 at 10,000 mA g^−1^ over 10,000 cycles	47%	/

**Fig. 4:**
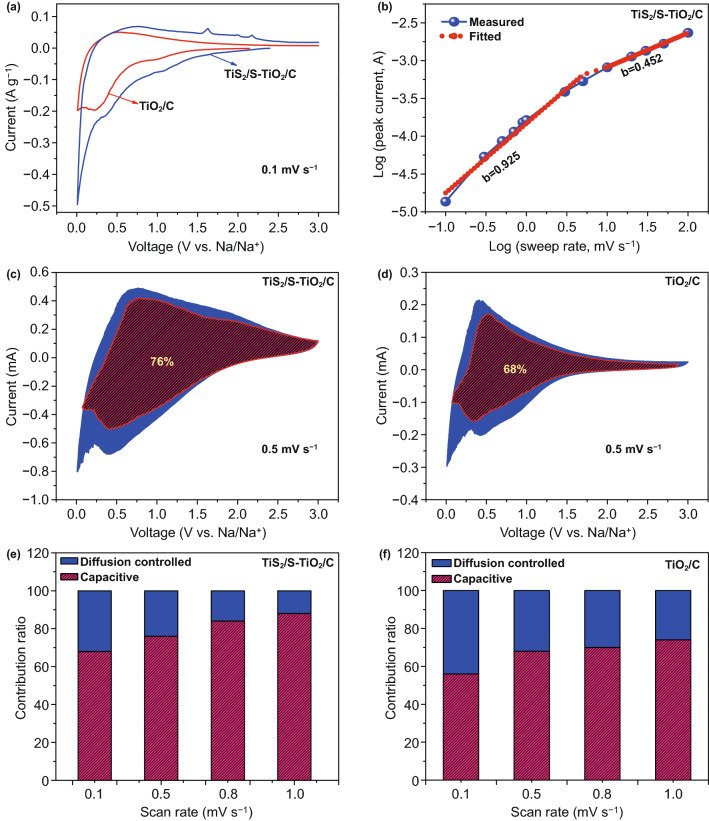
**a** CV curve of the first cycle at 0.1 mV s^−1^. **b** Relationship between log(i) and log(v). **c**, **d** Pseudo-capacitive contribution at 0.5 mV s^−1^. **e**, **f** The ratio of pseudo-capacitive contribution (pink) at various scan rates of electrochemical impedance spectroscopy plots of TiS_2_/S-TiO_2_/C nanofibers electrode and TiO_2_/C nanofibers electrode in SIBs

To get a deeper insight into the electrochemical behavior of the TiS_2_/S-TiO_2_/C electrode, representative cyclic voltammetry (CV) measurements were carried out at a scan rate of 0.1 mV s^−1^ in the range of 0.01–3.0 V (vs Na/Na^+^). As illustrated in Fig. 4a, for two electrodes, the peak at 1.0–1.2 V is only observed in the first cathodic scan, which is ascribable to a typically irreversible reaction associated with the decomposition of the electrolyte and the formation of a solid electrolyte interface (SEI). A couple of broad redox peaks that appeared below 1.5 V suggest that the reversible reaction of Ti^3+/^Ti^4+^ takes place during sodium insertion/extraction into/out of the crystal structure of TiO_2_ [37–39]. At high voltages of 1.5–3.0 V, the TiS_2_/S-TiO_2_/C electrode shows one reductive peak (1.60 V) and two oxidative peaks (− 1.6 and 2.2 V) in the following four scans (Figs. 4a and S6a), corresponding to multiple reversible phase transitions during sodiation/desodiation of TiS_2_. This observation is in good agreement with the behavior of the reported pure TiS_2_ electrode, being indicative of the formation of Na_0.22_TiS_2_, Na_0.55_TiS_2_, or NaTiS_2_ phase [36]. It is notable that when TiS_2_ and S are incorporated, the composite electrodes show a small increase in the area of CV curves above 1.5 V, but a significant increase in the area of closed curves below 1.5 V. The results indicate not only that the enhancement of sodium storage of TiS_2_/S-TiO_2_/C nanofibers may originate from the storage contribution of incorporated TiS_2_, but also show the significance for the synergistic improvement of the electrochemical activity of TiO_2_. In order to gain further insight into the synergistic influences of TiS_2_ modification and S doping, a kinetic analysis on the basis of CV at various scan rates ranging from 0.1 to 100 mV s^−1^ is evaluated (Figs. S6b-c and S7a-c). In general, the reaction kinetics mechanism is divided into the two typical types of (i) diffusion-controlled process and (ii) capacitive-controlled behavior, whereby the former refers to the faradaic redox reaction from sodium-ion intercalation and the latter indicates surface faradaic pseudo-capacitive as well as non-faradaic double-layer contributions. The charge storage mechanism inferred from the parameter b is the relation (i = av^b^) (a and b are adjustable parameters) between peak current response (i) and the scan rates (v) [40]. When b, i.e., the slope of log(i) − log(v) plot, is close to 0.5 (b = 0.5), diffusion-controlled faradaic intercalation is suggested. The value being close to 1 (b = 1) signifies pseudo-capacitive storage kinetics. Figure 4b displays the log(i) − log(v) plots of the TiS_2_/S-TiO_2_/C electrode, where obviously b changes from 0.925 (at scan rates of 0.1 to 1 mV s^−1^) to 0.452 (at scan rates of 3 to 100 mV s^−1^). Obviously, the sodium storage mechanism of TiS_2_/S-TiO_2_/C is dominated by surface pseudo-capacitive behavior at low rates and then turns to a diffusion-controlled process at fast scans, which is in coincidence with the pseudo-capacitive storage phenomenon of T-Nb_2_O_5_/Li cell by Dunn et al. [41].

Furthermore, the ratios of the pseudo-capacitive storage capacity contribution in the total charge storage are calculated from *i*(V) = *k*_1_v + *k*_2_v^1/2^ [42]. Here, the quantitative indicators of the pseudo-capacitive and diffusive contributions are k_1_ and k_2_, which can be obtained on the basis of the current response at a particular voltage. As can be seen in Fig. 4c, at a scan rate of 0.5 mV s^−1^ the ratio of the pseudo-capacitive contribution for the TiS_2_/S-TiO_2_/C electrode is as high as 76%, which is higher than that of the TiO_2_/C electrode (Fig. 4d). Additionally, as indicated by the shadowed area shown in Figs. 4e and S6d-f, at low scan rates ranging from 0.1 to 1 mV s^−1^, the pseudo-capacitive contribution gradually increases from 68 to 88% with increasing scan rates. Similarly, at a given scan rate, the pseudocapacitance rates of the TiO_2_/C electrode are also calculated, exhibiting a lower surface pseudo-capacitive contribution (Figs. 4f and S7d-f). Such observation demonstrates the enhancement of the pseudo-capacitive contribution as a result of the introduction of TiS_2_ and S doping, similar to the reported pseudo-capacitive MoO_2_-modified TiO_2_ composites [43] and doped TiO_2_ materials [20, 44]. Here, the combination of pseudo-capacitive TiS_2_ and sulfur doping can further increase the pseudo-capacitive contribution to the total capacity.

To further deeply the influence of sulfur doping and TiS_2_ modification, the sodium-ion diffusion coefficient has been investigated according to a cyclic voltammetry (CV) method, with the basis of the following equation: *I*_p_ = Δ0.4463zFA(zF/RT)^1/2^Δ*C*_0_*D*_Na_^1/2^v^1/2^ [45]. Here, *R* is the gas constant, *T* is the absolute temperature, Δ*C*_0_ is the surface concentration of the electrode material, while *I*_p_ and *v* represent peak current and scan rate, respectively. The sodium-ion diffusion coefficient can be determined by the peak current (*I*_p_) versus *v*^1/2^ based on the Randles–Sevcik equation. The apparent sodium diffusion coefficients of the TiS_2_/S-TiO_2_/C electrode are calculated to be 8.34 × 10^−10^ cm^2^ s^−1^, which is higher than D_Na_ values of TiO_2_/C electrode (5.82 × 10^−10^ cm^2^ s^−1^) without any sulfur content (Fig. S9). It suggests that the D_Na_ of the TiS_2_/S-TiO_2_/C electrode can be further improved by the synergistic effect of sulfur dopant and TiS_2_ modification.

Such pronounced pseudo-capacitive sodium storage mechanism could be related to the combination of nanostructured and pseudo-capacitive components of TiS_2_/S-TiO_2_/C composites electrode. The nanometer-sized TiS_2_/TiO_2_ composites can greatly improve the ion diffusivity and enhance the density at interfacial storage sites in the surface/near surface. The measured pseudo-capacitive storage behavior allows to a fast uptake/release of the sodium ion and long cycle life at ultrahigh rate for TiS_2_/S-TiO_2_/C electrodes.

## Conclusions

In summary, we developed a TiS_2_/S-TiO_2_/C composite as a superior potential electrode for SIBs with astonishing high-rate capacities and long-term cycling stabilities. The TiS_2_/S-TiO_2_ nanoparticles embedded in carbon nanofibers are obtained through electrospinning followed by a calcination in H_2_S gas. The decoration of TiO_2_ by TiS_2_ and by sulfur doping, together with the integration into a carbon nanofibers framework, greatly improves the pseudo-capacitive behavior. As a result, the as-spun TiS_2_/S-TiO_2_/C electrode exhibits a reversible capacity of 274 mAh g^−1^ at 100 mA g^−1^ for over 400 cycles. Even at a high rate of 10,000 mA g^−1^ after 10,000 cycles, it still maintains a capacity of 58 mA g^−1^ without noticeable fading.

## Electronic supplementary material

Below is the link to the electronic supplementary material.

Supplementary material 1 (PDF 2014 kb)
