# Supplementary material for: Enhanced Pseudo-Capacitive Contributions to High-Performance Sodium Storage in TiO2/C Nanofibers via Double Effects of Sulfur Modification
Source: Nanomicro Lett. 2020 Aug 14;12:165. doi: 10.1007/s40820-020-00506-1 (PMC7770798; doi:10.1007/s40820-020-00506-1)
Supplement: Supplementary file 1 — Supplementary material 1 (PDF 2014 kb) [file 40820_2020_506_MOESM1_ESM.pdf]

Supporting Information for

Enhanced Pseudo-Capacitive Contributions to High-Performance Sodium Storage in TiO_2/C Nanofibers via Double Effects of Sulphur Modification

Yan Zhang[1], Yuanye Huang[1], Vesna Srot[1], Peter A. van Aken[1], Joachim Maier[1], Yan Yu[2, 3, *]

[1]Max Planck Institute for Solid State Research, Stuttgart 70569, Germany

[2]Hefei National Laboratory for Physical Sciences at the Microscale，Department of Materials Science and Engineering, Key Laboratory of Materials for Energy Conversion, Chinese Academy of Sciences (CAS), University of Science and Technology of China, , Hefei, Anhui, 230026, People's Republic of China

[3]Dalian National Laboratory for Clean Energy (DNL), Chinese Academy of Sciences (CAS), People's Republic of China

*Corresponding author. E-mail: yanyumse@ustc.edu.cn (Yan Yu)

Supplementary Figures

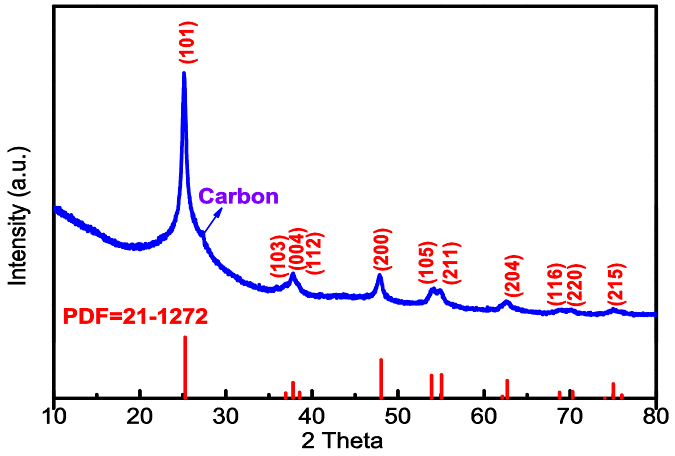

Fig. S1 XRD patterns of as-spun of TiO_2/C nanofiber composites

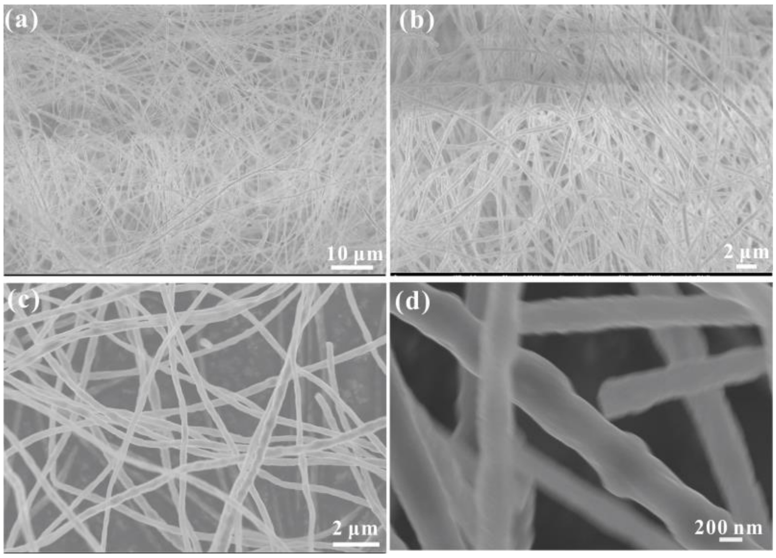

Fig. S2 SEM images of **a-b** as-spun PAN-TBOT precursors. **c-d** TiO_2/C nanofiber composites

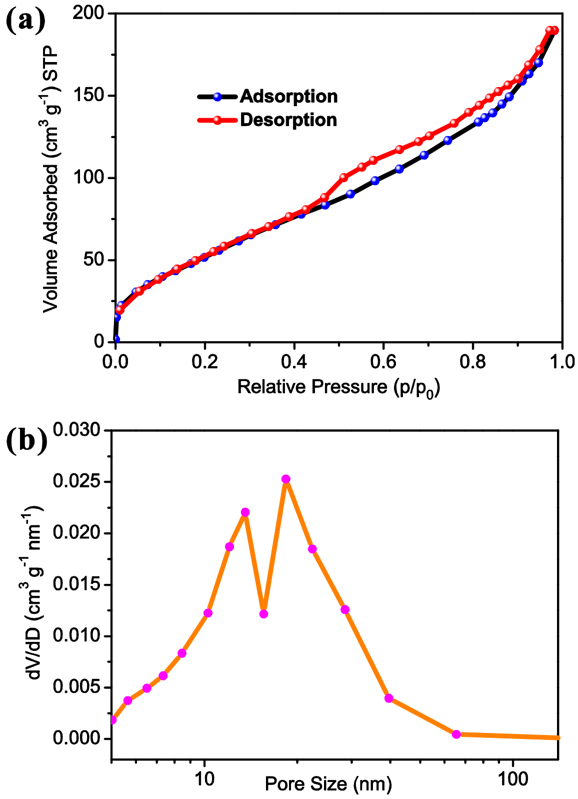

Fig. S3 a N_2 adsorption/desorption isotherm curves. **b** Corresponding DFT pore size distribution curves of $TiS_2/S\text{-}TiO_2/C$ nanofibers composites

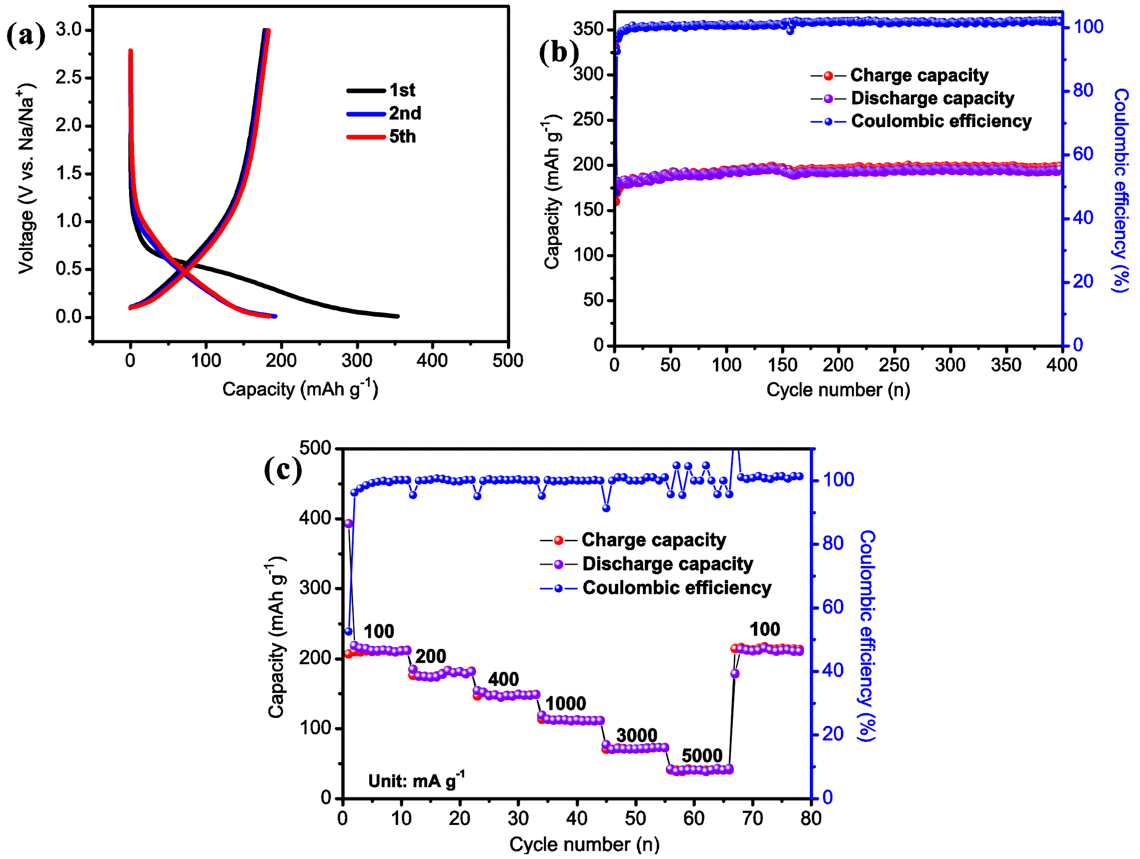

Fig. S4 a Charge-discharge profiles of the first, second and fifth cycle at a current density of 100 mA g^{-1}. **b** Cycling performances at a current density of 100 mA g^{-1}. **c** Rate performances at different densities of TiO_2/C nanofiber electrode in SIBs

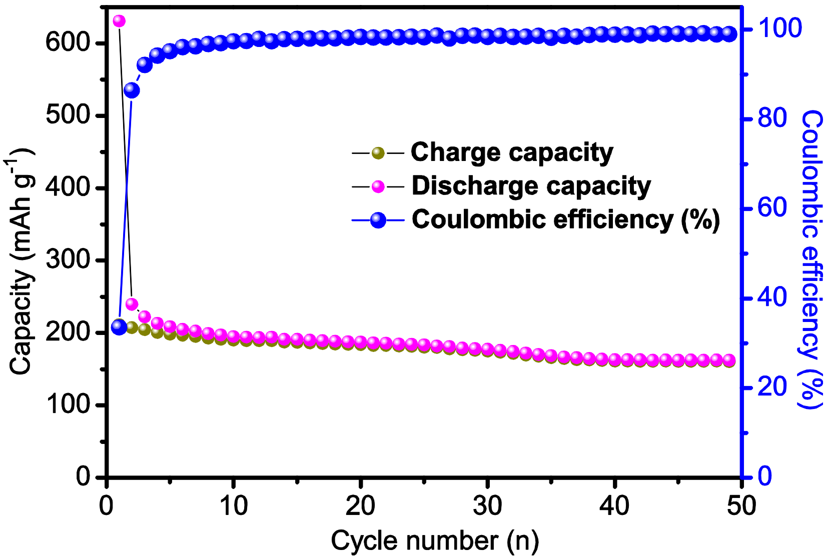

Fig. S5 Cycling performances at a current density of 100 mA g^{-1} of pure C nanofiber electrode in SIBs

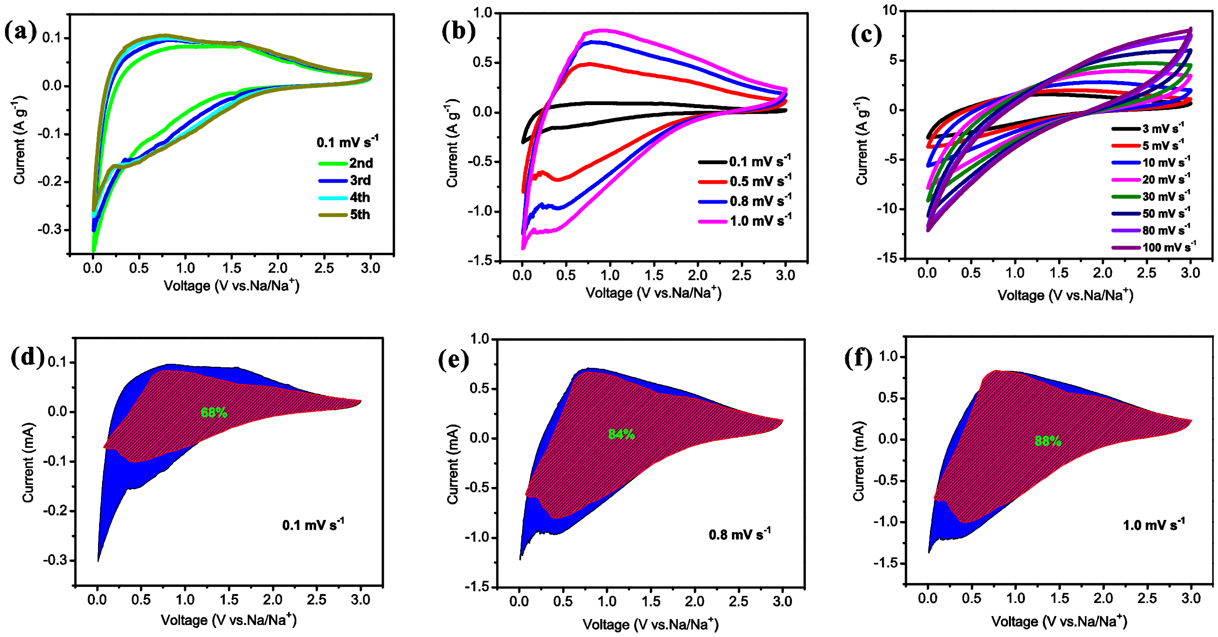

Fig. S6 a 2nd-5th CV curves at a scan rate of 0.1 mV s^{-1}. **b-c** CV curves at stepwise scan rates. **d-f** the capacitive contribution at a scan rate of 0.1 mV s^{-1}, 0.8 mV s^{-1} and 1.0 mV s^{-1} of TiS_2/S-TiO_2/C nanofiber electrode in SIBs

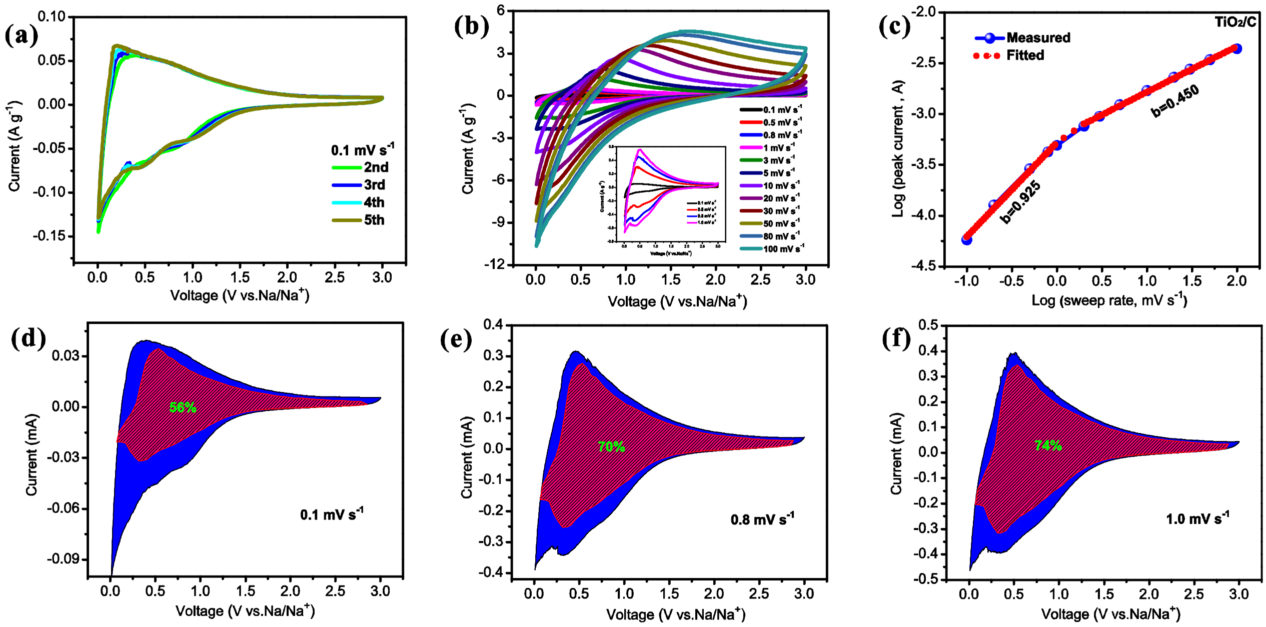

Fig. S7 a 2nd-5th CV curves at a scan rate of 0.1 mV s^{-1}. **b** CV curves at stepwise scan rates. **c** Relationship between log(i) and log(v). **d-f** Capacitive contribution at a scan rate of 0.1, 0.8, and 1.0 mV s^{-1} of TiO_2/C nanofiber electrode in SIBs

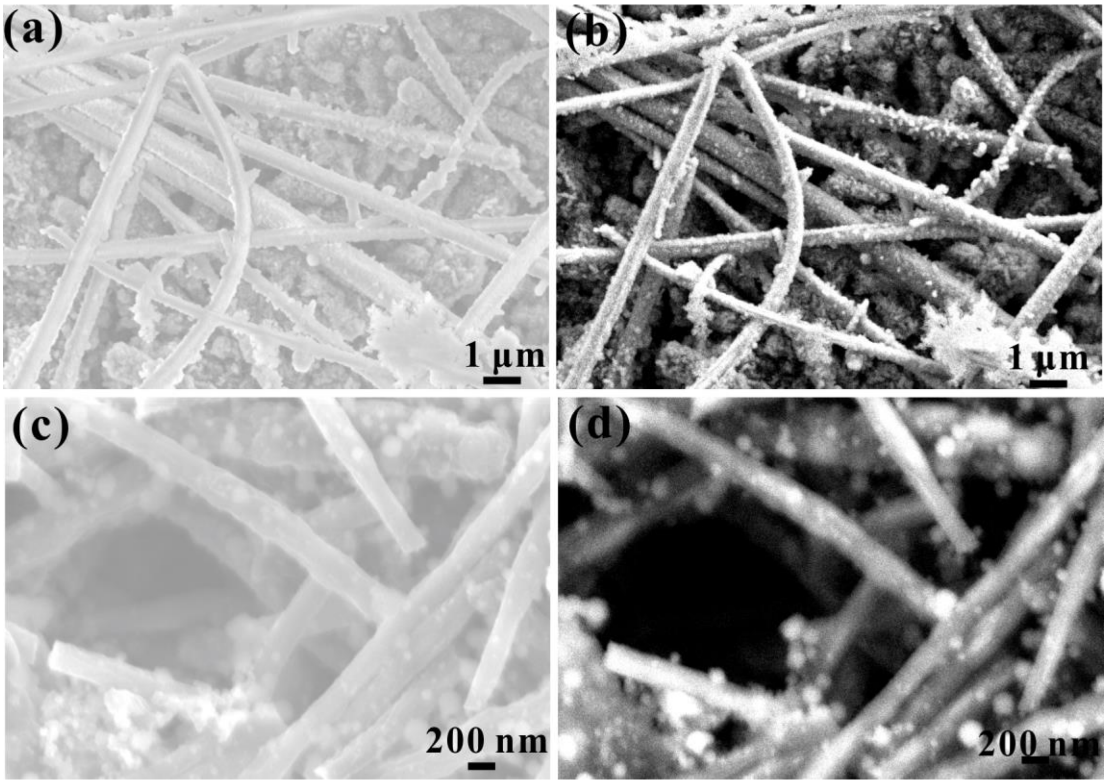

Fig. S8 SEM images of **a-b** TiS_2/S-TiO_2/C nanofibers electrodes and **c-d** TiO_2/C nanofiber electrodes after cycling

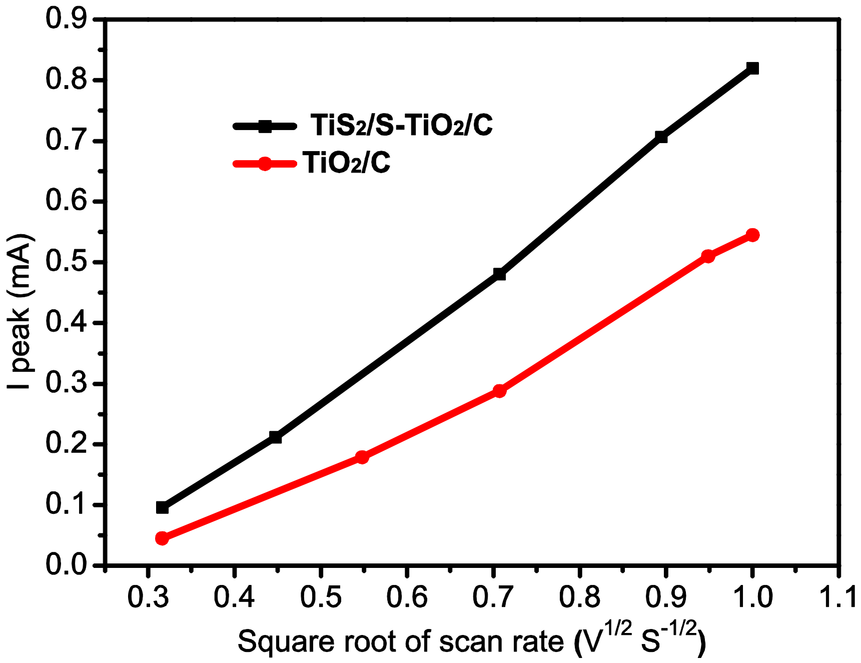

Fig. S9 Comparison of sodium ion diffusion coefficients for the TiS_2/S-TiO_2/C nanofibers electrodes and TiO_2/C nanofiber electrodes
